# Characterisation of Patients Receiving Moxifloxacin for Acute Bacterial Rhinosinusitis in Clinical Practice: Results from an International, Observational Cohort Study

**DOI:** 10.1371/journal.pone.0061927

**Published:** 2013-04-23

**Authors:** Ralph Mösges, Martin Desrosiers, Pierre Arvis, Stephanie Heldner

**Affiliations:** 1 University Hospital Cologne, Cologne, Germany; 2 Centre Hospitalier de l’Université de Montréal, Montreal, Canada; 3 Bayer Healthcare, Loos, France; 4 Bayer Pharma AG, Berlin, Germany; Hospital of the University of Pennsylvania, United States of America

## Abstract

We conducted a prospective, non-controlled, multi-centre Phase IV observational cohort study of patients with acute bacterial rhinosinusitis who were treated with moxifloxacin in clinical practice in 19 countries in Asia Pacific, Europe and the Middle East. With the data collected we evaluated the presentation and course of the current disease episode, particularly in terms of the principal clinical signs and symptoms of acute rhinosinusitis and diagnostic procedures. A final assessment of moxifloxacin therapy was made to evaluate the impact of the sinusitis episode on activities of daily life and on sleep disturbance, and to evaluate the clinical outcome of treatment. A total of 7,090 patients were enrolled, of whom 3909 (57.6%) were included in the valid for clinical outcome and safety population. Regional differences were observed in the main symptoms of acute rhinosinusitis and, according to several characteristics, disease episodes appeared to be more severe in patients in Europe than in the Asia Pacific or Middle East regions. The sinusitis episode impacted on daily living for mean (SD) periods of 3.6 (3.2), 4.6 (3.9) and 3.1 (3.0) days and disturbed sleep for 3.6 (3.2), 4.6 (3.9) and 3.1 (3.0) nights in the Asia Pacific, Europe and Middle East regions, respectively. With moxifloxacin treatment, the mean (SD) time to improvement of symptoms was 3.0 (1.5), 3.4 (1.6) and 3.2 (1.5) days, and the time to resolution of symptoms was 4.8 (2.6) days, 5.7 (2.4) days and 5.5 (2.5) days, in the Asia Pacific, Europe and Middle East regions, respectively. In conclusion, acute rhinosinusitis remains a substantial health burden with significant impact on patients’ quality of life, and there are differences between global regions in the clinical presentation, diagnosis and clinical course of disease episodes. Moxifloxacin was an effective and well-tolerated treatment option in the overall population.

***Registration:*** ClinicalTrials.gov Identifier: NCT00930488

## Introduction

Acute rhinosinusitis is a heterogeneous group of disorders in which inflammation of the mucosa of the paranasal sinuses and nose produce a variety of symptoms, including nasal blockage, obstruction and congestion, with or without facial pain or impaired smell. Rhinosinusitis is a major health problem, with substantial impact on quality of life, healthcare resources and spending, and productivity. Different subtypes of rhinosinusitis can be identified, based on patient history and a limited physical examination: acute, recurrent acute, and chronic. Acute rhinosinusitis is defined as the sudden onset of two or more symptoms, one of which should be either nasal blockage/obstruction/congestion or nasal discharge (anterior/posterior nasal drip), ± facial pain/pressure, ± reduction or loss of smell for less than 12 weeks according to some clinical guidelines [1], [2] or for 4 weeks or less as defined by others [3]–[5].

Mucosal inflammation in acute rhinosinusitis is often associated with viral or bacterial infection. Because the clinical features of such infections are similar, these two underlying causes are difficult to differentiate without invasive sinus-puncture studies [1]. In addition, many patients with symptoms of rhinosinusitis do not seek medical help [1] and the majority of cases resolve spontaneously [6]. Consequently, accurate epidemiological data for acute rhinosinusitis is difficult to obtain. Acute rhinosinusitis due to viral infection, such as the common cold, has been estimated to occur 2–5 times per year in an adult [1]. Non-bacterial rhinosinusitis may predispose a patient to a secondary bacterial sinus infection [7]. Bacterial superinfection of mucosa damaged by viral infection is an important cause of acute rhinosinusitis [1]. Positive bacterial culture tests have been found in 60% of acute rhinosinusitis cases with symptoms of upper respiratory tract infection for 10 days or more [8].

In recent years, a number of expert panels have produced evidence-based guidelines for the diagnosis and management of rhinosinusitis [1]–[5], [9], [10]. These guidelines are consistent in recognising that an assessment of symptoms and their severity is important to define the extent of disease and guide the selection of treatment aimed at reducing mucosal inflammation, controlling infection and restoring mucociliary clearance within the sinuses. A fundamental issue in determining appropriate treatment is the identification of cases for which antibiotics are appropriate.

Although the general presentation of viral and bacterial acute rhinosinusitis can be highly similar, the duration, pattern and/or severity of symptoms provide one of the simplest and generally reliable means of differentiating bacterial from viral illness. Symptoms of acute viral rhinosinusitis typically peak within 2–3 days of onset, decline gradually and then disappear within 10–14 days. Thus, a bacterial sinus infection is more likely if these symptoms worsen after 5–7 days or do not improve after 10–14 days [11].

Clinical guidelines generally agree that symptoms persisting for 10 days or more and/or showing a pattern of initial improvement followed by worsening are likely bacterial in origin. Similarly, most of the current guidelines suggest that unusually severe symptoms (e.g. high fever, unilateral facial/tooth pain, orbital cellulitis, intracranial expansion), particularly during the first several days of disease, are also suggestive of acute bacterial rhinosinusitis (ABS) [2]–[5], [10].

Confirmation of a bacterial aetiology is ordinarily not attained in routine clinical practice, since this requires antral puncture, or at least endoscopic sampling of the middle meatus [12]. Consequently, the choice of antibiotic therapy is empiric, in most cases, with selection depending on the probable infecting upper respiratory pathogens, bacterial antibiotic resistance and antibiotic pharmacological profiles. Numerous studies of antibiotic treatment of ABS have been published, many of which support the clinical benefit of antibiotic treatment. In addition, recent meta-analyses have confirmed that antibiotics provide a clear, albeit small benefit in hastening symptom relief compared with placebo, with different agents being similarly effective [10], [13], [14]. Despite this, antibiotic treatment of acute bacterial rhinosinusitis is controversial with some reports questioning whether such therapies benefit patients [15]–[18].

Amoxicillin or penicillin is generally recommended as the first-line antibiotic treatment for adults with ABS, substituted with trimethoprim-sulphamethoxazole or a macrolide antibiotic for patients with β-lactam allergy [5], although in various parts of the world these antibiotics are not recommended as second line therapies any longer due to high resistance rates. Second-line therapies include amoxicillin-clavulanate, the respiratory fluoroquinolones (moxifloxacin, levofloxacin and gatifloxacin) and the second generation cephalosporins (cefuroxime, cefpodoxime, cefprozil or cefdinir) [9], [19], [20]. However, among these agents gatifloxacin has been withdrawn from the market for systemic use due to serious side effects (hyperglycaemia as well as hypoglycaemia) [21].

The pharmacokinetic and antibacterial characteristics of moxifloxacin support its use in acute bacterial rhinosinusitis [22], [24] and previous studies have shown that treatment with moxifloxacin rapidly improves the signs and symptoms of affected patients [25], [26]. According to several clinical guidelines, this respiratory fluoroquinolone is an appropriate choice of antibiotic in selected cases, such as those with more severe disease, those with a history of β-lactam allergy, where first-line antibiotic treatment has failed, where resistance to first-line antibiotics is suspected or in patients for whom the consequences of treatment failure could be serious [9], [10], [27].

The two main objectives of this observational study (ClinicalTrials.gov identifier: NCT00930488) were 1) to collect data on the characteristics of patients with ABS, the history and frequency of rhinosinusitis episodes, and the diagnostic procedures and therapeutic options chosen by investigators and 2) to evaluate the potential benefits of antibacterial therapy with moxifloxacin in patients with ABS to whom this treatment was prescribed in clinical practice. Thus, the study sought to improve the current understanding of this common disease and its routine clinical management in different regions of the world.

## Methods

The STROBE checklist relevant to this trial is available as supporting information; see Checklist S1.

### Study Design

This was a prospective, non-controlled, multi-centre, observational, Phase IV cohort study conducted in 19 countries in three global regions: Asia Pacific (China, Indonesia, Malaysia, Pakistan, the Philippines and Singapore); Europe (Austria, France, Germany, The Netherlands and Romania); and the Middle East (Bahrain, Egypt, Jordan, Kuwait, Lebanon, Saudi Arabia, United Arab Emirates, Yemen).

### Setting

The majority of investigators were ear, nose and throat (ENT) physicians. Investigators were proposed by the study sponsor’s local representative. Within each country, as many sites as possible were included to ensure sampling was nationally representative.

### Participants

The study included patients with an acute bacterial rhinosinusitis diagnosed by their attending physician and who were suitable for treatment with moxifloxacin, based on the relevant Summary of Product Characteristics (SmPC). The diagnosis of ABS was made at the discretion of the attending investigator, according to his or her medical practice; a definition was not given. Patients were excluded from participation as contraindicated by the SmPC.

### Treatments and Follow-up

The dosage and duration of moxifloxacin treatment was as decided by the treating physician. The study protocol recommended that this should follow the SmPC in each country, which in most cases was moxifloxacin 400 mg q.d. for 7 days. The observation period for each patient covered at least the duration of the treatment period with moxifloxacin. After the initial clinic visit when moxifloxacin treatment was started, patients returned for a first and potentially second follow-up visit, the latter being decided at the discretion of the attending physician.

### Data Collection

For each patient, a standardised case report form (CRF) was used to record data for patient characteristics, the history of rhinosinusitis, the last episode of rhinosinusitis, concomitant diseases and details on the current episode of acute rhinosinusitis. For the current episode, diagnostic measures, purulence and microbiology (if tested; confirmation of bacterial aetiology was not required), the sinuses involved, the duration of symptoms before the start of antibiotic treatment and prior antibiotic use were recorded. At each clinic visit, the treating physician recorded the patient’s general condition, the severity of rhinosinusitis and the presence and severity of the following clinical signs and symptoms: fever, nasal obstruction, nasal secretion, post-nasal secretion, pain or pressure and hyposmia. At the last clinic visit after moxifloxacin treatment, a final assessment of therapy was made to evaluate: the duration of treatment until improvement; the duration of treatment until symptom-free; the impact of the episode on activities of daily life and on sleep disturbance; and, based on the physician’s overall impression, the overall clinical outcome and tolerability of treatment.

The patient’s general condition was classified as “good”, “fair” or “serious” and the severity of rhinosinusitis as “no infection” (except at the start of moxifloxacin treatment), “mild”, “moderate” or “severe”. A symptom (severity of rhinosinusitis, fever, nasal obstruction, post nasal secretion, pain or pressure, hyposmia) was categorised as “relieved” if it was assessed as “none” or “no infection” at the last follow-up visit, as “improved” if it was assessed with a better category at the last follow-up visit compared to the start of therapy, as “unchanged” if assessments did not change, and as “worsened” if it was assessed with a worse category at the last follow-up visit compared to the start of therapy. The course of a patient’s general condition was categorised as “improved” if the patient’s general condition was better at the last follow-up visit compared to the start of therapy, as “unchanged” if it did not change and as “worsened” if it was worse at the last follow-up visit compared to the start of therapy.

Any adverse events (AEs) experienced after the start of moxifloxacin treatment were recorded in all patients. An AE was defined as any unfavourable and/or unintended sign (including a clinically significant change in laboratory or ECG findings), symptom or disease. AEs were summarised using the Medical Dictionary for Drug Regulatory Activities (MedDRA) and categorised according to relationship to treatment, seriousness, discontinuation of treatment and outcome. Incidences of AEs were calculated based on both the number of patients and the number of events.

### Ethical Declarations

The study was conducted in accordance with the Declaration of Helsinki and Good Clinical Practice guidelines, European Medicines Agency and US Food and Drug Administration guidelines and applicable local law(s) and regulation(s). Where required, approval for the study was obtained from local responsible authorities (ethics committees and/or institutional review boards) before study start. Additional investigations beyond the investigators’ regular practice were not performed and patients were not randomised to any treatment. Moxifloxacin was prescribed for an approved indication within the regular practice of the treating physician independent of his/her participation in this study.

Where required, patients were informed about the study and a written consent was obtained at the start of the study. Patients could be withdrawn at any time without giving reasons and without consequences for medical care.

### Analyses and Analysis Populations

Analyses were performed on the valid for clinical outcome and safety population which comprised all patients who took at least one dose of moxifloxacin and had sufficient follow-up information for the approved indication on clinical outcome and safety. The final valid for clinical outcome and safety population presented in this manuscript excluded patients with an acute exacerbation of chronic sinusitis. Chronic sinusitis was defined as the presence of signs and symptoms for longer than 12 weeks based on previous guidelines [1]–[5]. Statistical analyses were exploratory and descriptive analysis of the data was performed using summary statistics for categorical and quantitative (continuous) data. Baseline characteristics and the effectiveness and tolerability of treatment were compared descriptively between patient subgroups defined by regions. Based on the sign and symptom assessments at start of therapy and at the last follow-up visit, calculations were performed for the course of patients’ general condition, severity of sinusitis, fever, nasal obstruction, nasal secretion, post-nasal secretion, pain or pressure, and hyposmia.

No statistical tests were performed because statistical tests in non-interventional and observational studies can only be used for exploratory purposes and not to test causalities. Percentages were calculated as the proportion of each category, including the category of missing values. Where specified, percentages were calculated based on documented (non-missing) values only.

### Registration

ClinicalTrials.gov Identifier: NCT00930488 http://clinicaltrials.gov/ct2/show/NCT00930488.

## Results

The study was run between 2^nd^ of March 2007 and 31^st^ of December 2008. A total of 883 investigators contributed to the study by returning at least one CRF and a total of 7090 patients were enrolled. Of this total population, 313 patients were excluded from the valid for clinical outcome and safety population for a variety of reasons, including: first visit before the study start (n = 70); patient received treatment with a different antibiotic (n = 67); treatment and documentation occurred after the study end (n = 58); no visits recorded (n = 52); all visits performed before the study start (n = 36); patient did not receive moxifloxacin (n = 33); and lost to follow-up (n = 24). A patient could have had more than one reason for exclusion. Thus, acute bacterial rhinosinusitis patients receiving at least one dose of moxifloxacin in the study period of no more than 2 days before study start in their respective country until official study end were considered valid for the clinical outcome and safety analyses. The number of valid patients (%) in the participating countries or regions were: Austria, 388 (5.7); China, 588 (8.7); the Middle East (the lead country for this region was Egypt and included the Gulf states), 2386 (35.2); France, 513 (7.6); Germany, 241 (3.6); Indonesia, 221 (3.3); Malaysia, 358 (5.3); Netherlands, 110 (1.6); Pakistan, 465 (6.9); the Philippines, 701 (10.3); Romania, 572 (8.4); and Singapore 234 (3.5).

### Patient Characteristics and Inclusion in the Clinical Outcome Analysis

**Figure 1:**
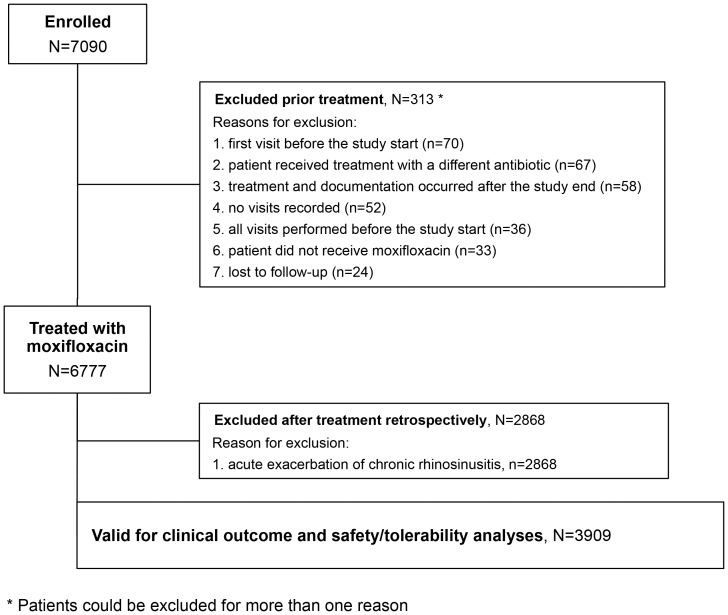
Flowchart of patient selection.

The demographic characteristics and disease history of the 3909 patients diagnosed with only acute rhinosinusitis are shown in Table 1. Additionally, 2868 patients (42.3%) suffered from underlying chronic rhinosinusitis. These patients, who were enrolled into the study after presenting with an acute exacerbation, were excluded from the final clinical outcome and safety analyses as it is not an approved indication for moxifloxacin (Figure 1).

**Table 1:** Patient baseline characteristics, disease history and impact of previous rhinosinusitis.

Characteristics		Total population (n = 3909)	Asia Pacific (n = 1421)	Europe (n = 1261)	Middle East (n = 1227)
Male, n (%)		2006 (51.3)	740 (52.1)	540 (42.8)	726 (59.2)
Age, mean (SD) years		39.9 (13.9)	39.5 (13.4)	44.1 (15.6)	35.9 (11.1)
Age group, years	>0–<20	150 (3.8)	47 (3.3)	47 (3.7)	56 (4.6)
	≥20–<40	1963 (50.2)	738 (51.9)	486 (38.5)	739 (60.2)
	≥40–<60	1371 (35.1)	517 (36.4)	505 (40.0)	349 (28.4)
	≥60–<80	344 (8.8)	105 (7.4)	203 (16.1)	36 (2.9)
	≥80	34 (0.9)	12 (0.8)	20 (1.6)	2 (0.2)
BMI, mean (SD) kg/m^2^		25.0 (4.4)	24.1 (4.5)	24.8 (4.2)	26.4 (4.0)
At least one rhinosinusitis episode, n (%)^a^		1353 (34.6)	465 (32.7)	419 (33.2)	469 (38.2)
Surgical treatment of rhinosinusitis, n (%)		99 (2.5)	22 (1.5)	45 (3.6)	32 (2.6)
Nights with sleep disturbance due to last rhinosinusitis episode, n (%)^a^		808 (20.7)	448 (31.5)	373 (29.6)	571 (46.5)
Days with impact on daily living due to last rhinosinusitis episode, n (%)^a^		949 (24.3)	518 (36.5)	511 (40.5)	593 (48.3)
At least one physician visit due to rhinosinusitis episode, n (%)^a^		1209 (30.9)	402 (28.3)	388 (30.8)	419 (34.1)
Purulent nasal secretions at diagnosis, n (%)		2995 (76.6)	1040 (73.2)	1063 (84.3)	892 (72.7)
Microbiological assessment, n (%)		276 (7.1)	82 (5.8)	113 (9.0)	81 (6.6)
Isolation of causative bacteria, n/N^b^ (%)		165/276 (59.8)	38/82 (46.3)	82/113 (72.6)	45/81 (55.6)

a In the last 12 months;

b N = number of patients with samples taken; BMI = body mass index; SD = standard deviation.

### Current Episode of Acute Rhinosinusitis

Involvement of all four paranasal sinuses in the infection was highest in patients in Europe. Acute rhinosinusitis involving the ethmoidal sinus was less common in patients in the Asia Pacific region than in those in European or Middle Eastern countries. Similarly, involvement of more than one sinus was generally more common in European patients, whereas isolated sinus involvement was more likely in Asia Pacific patients (Table 2). The majority of the patients had maxillary sinusitis (84.3%) followed by frontal sinusitis in approximately 40% of the patients. In addition, a small percentage of patients (4.4%) were diagnosed with sphenoidal sinusitis; these individuals had an average of three sinuses (3.1±1.1) involved in the disease.

**Table 2:** Current episode of rhinosinusitis: signs, symptoms and medication.

Characteristics or symptoms at start of therapy			Total population (n = 3909)	Asia Pacific (n = 1421)	Europe (n = 1261)	Middle East (n = 1227)
Sinus involved	Maxillary		3294 (84.3)	1149 (80.9)	1104 (87.5)	1041 (84.8)
	Frontal		1533 (39.2)	515 (36.2)	557 (44.2)	461 (37.6)
	Ethmoidal		1456 (37.2)	426 (30.0)	515 (40.8)	515 (42.0)
	Sphenoidal		172 (4.4)	58 (4.1)	67 (5.3)	47 (3.8)
Number of involved sinuses	1		1838 (47.0)	825 (58.1)	492 (39.0)	521 (42.5)
	2		1467 (37.5)	423 (29.8)	569 (45.1)	475 (38.7)
	3		449 (11.5)	123 (8.7)	163 (12.9)	163 (13.3)
	4		84 (2.1)	27 (1.9)	31 (2.5)	26 (2.1)
Sinus signs and symptoms	Nasal obstruction^a^	Mild	909 (23.3)	464 (32.7)	170 (13.5)	275 (22.4)
		Moderate	1993 (51.0)	701 (49.3)	628 (49.8)	664 (54.1)
		Severe	811 (20.7)	176 (12.4)	423 (33.5)	212 (17.3)
	Nasal secretions^a^	Clear	329 (8.4)	166 (11.7)	55 (4.4)	108 (8.8)
		Mucoid	1019 (26.1)	410 (28.9)	274 (21.7)	335 (27.3)
		Purulent	2301 (58.9)	753 (53.0)	861 (68.3)	687 (56.0)
	Post-nasal secretions^a^	Mild	1213 (31.0)	547 (38.5)	283 (22.4)	383 (31.2)
		Moderate	1799 (46.0)	600 (42.2)	646 (51.2)	553 (45.1)
		Severe	465 (11.9)	88 (6.2)	220 (17.4)	157 (12.8)
	Pain/pressure^a^	Mild	1246 (31.9)	599 (42.2)	276 (21.9)	371 (30.2)
		Moderate	1639 (41.9)	488 (34.3)	619 (49.1)	532 (43.4)
		Severe	520 (13.3)	96 (6.8)	258 (20.5)	166 (13.5)
	Hyposmia^a^	Mild	1382 (35.4)	537 (37.8)	434 (34.4)	411 (33.5)
		Moderate	928 (23.7)	225 (15.8)	427 (33.9)	276 (22.5)
		Severe	253 (6.5)	61 (4.3)	136 (10.8)	56 (4.6)
	Fever (°C)	Mild (37.5–38.0)	1313 (33.6)	500 (35.2)	385 (30.5)	428 (34.9)
		Moderate (38.1–39.0)	985 (25.2)	297 (20.9)	329 (26.1)	359 (29.3)
		Severe (>39.0)	102 (2.6)	28 (2.0)	42 (3.3)	32 (2.6)
Patient condition^a^	Good		749 (19.2)	269 (18.9)	301 (23.9)	179 (14.6)
	Fair		2596 (66.4)	1017 (71.6)	711 (56.4)	868 (70.7)
	Serious		506 (12.9)	120 (8.4)	230 (18.2)	156 (12.7)
Severity of rhinosinusitis^a^	Mild		555 (14.2)	275 (19.4)	80 (6.3)	200 (16.3)
	Moderate		2463 (63.0)	953 (67.1)	805 (63.8)	705 (57.5)
	Severe		831 (21.3)	177 (12.5)	354 (28.1)	300 (24.4)
Number of severe symptoms per patient	None		1277 (32.7)	579 (40.7)	268 (21.3)	430 (35.0)
	1		1487 (38.0)	594 (41.8)	430 (34.1)	463 (37.7)
	2		605 (15.5)	152 (10.7)	287 (22.8)	166 (13.5)
	3		298 (7.6)	53 (3.7)	149 (11.8)	96 (7.8)
	4		130 (3.3)	25 (1.8)	73 (5.8)	32 (2.6)
	5		43 (1.1)	3 (0.2)	25 (2.0)	15 (1.2)
	6		21 (0.5)	5 (0.4)	12 (1.0)	4 (0.3)
Concurrent medications, n (%)	Topical decongestants or nasal preparations		1488 (38.1)	338 (23.8)	577 (45.8)	573 (46.7)
	Systemic nasal decongestants		307 (7.9)	154 (10.8)	59 (4.7)	94 (7.7)
	Decongestants and anti-allergics		605 (15.5)	98 (6.9)	246 (19.5)	261 (21.3)
Comorbidities, n (%)			2282 (58.4)	810 (57.0)	780 (61.9)	692 (56.4)
Respiratory, thoracic, mediastinal, n (%)			1425 (36.4)	534 (37.6)	394 (31.2)	497 (40.5)
Metabolism, n (%)			301 (7.7)	113 (8.0)	98 (7.8)	90 (7.3)
Cardiac, n (%)			291 (7.4)	100 (7.0)	140 (11.1)	51 (4.2)
Ear, n (%)			284 (7.3)	89 (6.3)	105 (8.3)	90 (7.3)
Previous antibiotic therapy for current episode, n (%)			882 (22.6)	370 (26.0)	275 (21.8)	237 (19.3)

a Graded by the investigator.

Regional differences were observed in the main symptoms of acute rhinosinusitis. Severe nasal obstruction, purulent nasal secretions, moderate or severe post-nasal secretions, moderate or severe sinus pain or pressure, and moderate or severe hyposmia were more frequent among patients in Europe than among those in the other two regions.

In the clinical outcome population overall, the duration of symptoms was (mean ± SD) 5.9±5.4 days before starting antibiotic treatment. Patients in Europe waited for a longer duration (6.8±6.0 days) than those in Asia Pacific countries (5.8±5.2 days) and the Middle East (4.8±3.8 days). A higher proportion of patients in the Asia Pacific region had failed previous antibiotic treatment for the study episode of acute rhinosinusitis than in the other two regions.

### Diagnostic Procedures used by Physicians

In addition to evaluation of clinical signs and symptoms, further diagnostic measures were performed to confirm the presence of acute rhinosinusitis with X-ray, rhinoscopy and endoscopy being carried out most frequently (Table 3). Approximately 60% of patients were diagnosed by the use of more than one diagnostic measure. In patients who underwent computed tomography (CT) scanning, sphenoidal sinusitis (and the number of sinuses involved) could be correctly diagnosed. This explains why the use of CT was more than twice as frequent with sphenoidal sinus involvement compared with other sinuses, and rhinoscopy was more likely to be used when the ethmoidal sinus was involved.

**Table 3:** Diagnostic measures used for current episode of rhinosinusitis.

Characteristic^a^	Total population (n = 3909)	Asia Pacific (n = 1421)	Europe (n = 1261)	Middle East (n = 1227)
Ultrasound	114 (2.9)	4 (0.3)	110 (8.7)	0 (-)
X-ray	1518 (38.8)	403 (28.4)	599 (47.5)	516 (42.1)
Computed tomography	509 (13.0)	174 (12.2)	127 (10.1)	208 (17.0)
Magnetic resonance tomography	23 (0.6)	3 (0.2)	14 (1.1)	6 (0.5)
Sinus puncture	151 (3.9)	24 (1.7)	120 (9.5)	7 (0.6)
Nasal swab	116 (3.0)	23 (1.6)	66 (5.2)	27 (2.2)
Rhinoscopy	1306 (33.4)	372 (26.2)	603 (47.8)	331 (27.0)
Endoscopy	808 (20.7)	169 (11.9)	429 (34.0)	210 (17.1)

a Patients could have ≥1 diagnostic measures.

Regional differences in diagnostic approach were noted, with use of X-ray, ultrasound, endoscopy, sinus puncture and rhinoscopy more likely in Europe compared with the other regions (Table 3). The mean number of diagnostic measures used per patient was 2.6 in Europe, 1.9 in the Middle East, and 1.7 in the Asia Pacific region.

Microbiological testing was carried out in 7.1% of the overall population, the majority (N = 2995, 76.6%) of whom had purulent nasal secretion at the time of diagnosis of rhinosinusitis. Causative pathogens were isolated from 59.8% of the obtained specimens.

### The Impact of Sinus Puncture on Diagnosis, Severity of Sinusitis and Patients’ Characteristics

Data from a subgroup analysis of patients with or without sinus puncture show that patients with sinus puncture received twice as many diagnostic tools (mean ± SD, 4.1±1.2 vs 2.0±1.0, respectively); they were more likely to have purulent nasal secretion (79.5% vs 58.0%, respectively), severe nasal congestion (36.4% vs 21.0%, respectively), severe or moderate pain (18.5% vs 13.1% and 66.9% vs 40.9%, respectively), moderate fever (50.3% vs 24.2%, respectively), hyposmia (60.9% vs 22.2%, respectively) and post-nasal secretion (65.6% vs 45.2%, respectively). Patients with sinus puncture were more likely to have moderate sinusitis (76.8% vs 62.5%, respectively) and patients were considered to be in “fair or severe” condition (86.1% vs 65.6% and 9.3% vs 13.1%, respectively). Patients with sinus puncture had slightly more comorbidities (mean ± SD, 1.4±2.1 vs 0.9±1.1, respectively). There was no difference in the number of sinuses involved in sinusitis among patients with or without sinus puncture (mean ± SD, 1.6±0.8 sinuses vs 1.7±0.8 sinuses, respectively). There was a great variation among the regions in the percentages of patients with sinus puncture (n = 151): most were performed in Europe (n = 120, 79.5%), followed by Asia (n = 24, 15.9%) and the Middle East (n = 7, 4.6%).

### Severity of Illness

The majority of patients were rated as having a ”fair” general condition and a ”moderate” sinusitis by their treating physician at the first clinic visit (Table 2). Patient condition varied across regions, with the highest proportion of patients judged to be in a ”serious” condition and having a “severe” rhinosinusitis in Europe, followed by the Middle East then Asia Pacific. Similarly, the frequency of severe rhinosinusitis was highest in Europe, followed by the Middle East and Asia. Patients in Europe were the least likely to present with mild rhinosinusitis. Conversely, the frequency of mild rhinosinusitis was highest in the Asia Pacific region. The pattern of patient condition and rhinosinusitis severity reflected the proportions of patients with 2 or more severe symptoms in each region.

### Comorbidities

Of the 3909 patients involved in the analysis, 2282 (58.4%) had at least one concomitant disease. They suffered in most of the cases from respiratory, thoracic or mediastinal diseases (1425, 36.4%) followed by metabolic (301, 7.7%), cardiac (291, 7.4%) and ear-related (284, 7.3%) diseases. There were regional variations in these comorbid conditions: more cardiac (11.1%) and less respiratory (31.2%) comorbidities were diagnosed in European patients than in Asia or in the Middle East (Table 2).

### Impact of Rhinosinusitis

Acute rhinosinusitis had an impact on the activities of daily life in the majority of patients (Table 4). The average proportion of patients affected was higher in Europe than in the Middle East or Asia Pacific regions and the mean duration of an impact on daily activities was markedly longer in patients in Europe than the other two regions. Compared with patients in Asia Pacific and Europe, those in the Middle East were more likely to experience sleep disturbances, but the duration of disturbances was shorter.

**Table 4:** Impact of current rhinosinusitis episode.

Characteristic	Total population (n = 3909)	Asia Pacific (n = 1421)	Europe (n = 1261)	Middle East (n = 1227)
Number of patients with impact on dailyliving, n (%)	2693 (68.9)	939 (66.1)	908 (72.0)	846 (68.9)
Impact on daily living, mean (SD) days	3.8 (3.5)	3.6 (3.2)	4.6 (3.9)	3.1 (3.0)
Nights with sleep disturbance, n (%)	2320 (59.4)	827 (58.2)	721 (57.2)	772 (62.9)
Sleep disturbance, mean (SD) nights	3.1 (3.0)	3.2 (3.2)	3.3 (3.3)	2.7 (2.4)

SD = standard deviation.

### Duration and Clinical Outcome of Moxifloxacin Treatment

**Figure 2:**
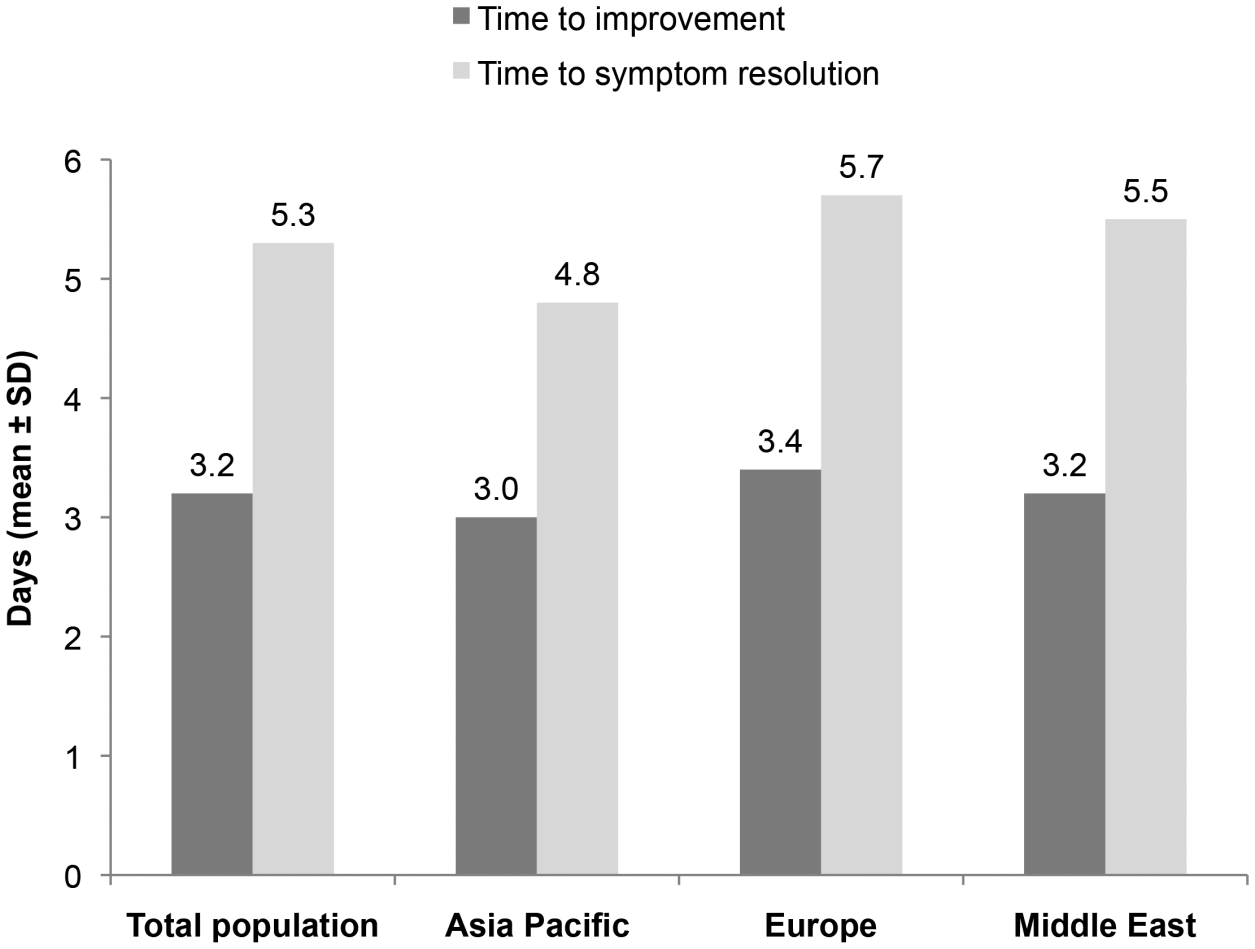
Recovery: Time to improvement of symptoms and time to symptom resolution.

Moxifloxacin treatment was administered for up to 21 days, and for a mean (SD) duration of 7.3 (1.8) days. Mean treatment duration and mean daily dose of moxifloxacin was similar across the three regions. With moxifloxacin treatment, the mean (SD) time to improvement of symptoms was 3.2 (1.5) days and it was 5.3 (2.6) days to resolution of symptoms (Figure 2). No marked differences were seen between recovery times in patients with mild or moderate *vs* severe disease, irrespective of the number of severe symptoms. Mean recovery times were also similar in different regions. Patients in the Asia Pacific region reported symptom resolution earlier on average than those in Europe or the Middle East.

Sixty-seven patients (1.7%) switched to an alternative antibiotic, with a slightly higher rate of switching in Europe (2.1%) compared with Asia Pacific (1.5%) or the Middle East (1.5%). The overall rate of premature discontinuation of moxifloxacin treatment was 2.5%, with small variation in rates in the different regions: Europe (1.8%), Asia Pacific (2.7%) and the Middle East (2.8%).

### Other Therapies

Additional therapies could also be provided for the patients in routine clinical practice. For example, in a small proportion of patients sinus irrigation (n = 323, 8.3%) and surgical drainage (n = 38, 1.0%) were applied. There was, however, a regional difference in the frequency of these techniques. These were most frequently applied in Europe (12.2% and 1.1%, respectively) and less frequently in Asia (7.5% and 1.1%, respectively) or in the Middle East (5.1% and 0.2%, respectively).

### The Effect of Prior Antibiotic Therapy on Diagnosis, Severity of Sinusitis and Patients’ Characteristics

Another subgroup analysis indicates that patients who were pre-treated with a different antibiotic compared with those without prior antibiotic therapy were more likely to have severe sinusitis at the time of diagnosis (27.7% vs 18.6%, respectively), reporting severe pain (16.4% vs 11.5%, respectively), nasal congestion (24.5% vs 19.0%, respectively), post-nasal secretion (16.8% vs 10.1%, respectively), moderate hyposmia (26.2% vs 22.8%, respectively) and purulent nasal secretion (64.3% vs 57.3%, respectively).

Patients who received a prior course of antibiotic therapy for the current episode were more likely to consult a physician in the previous 12 months (33.8% vs 18.3%, respectively) and the last sinusitis episode was more likely to occur in the previous 3 months (33.5% vs 17.2%, respectively). Among patients who reported that sinusitis had an impact on their daily life, the duration of this time period was 1.5 days longer in those with prior antibiotic therapy versus without prior antibiotic therapy (mean ± SD, 6.2±6.1 days vs 4.7±5.0 days, respectively).

There was no impact of the prior antibiotic therapy versus no prior antibiotic therapy on the length of time until patients experienced improvement in their symptoms (mean ± SD, 3.4±1.8 vs 3.2±1.5, respectively) or they became symptoms-free (mean ± SD, 5.8±3.0 vs 5.2±2.4, respectively) during treatment with moxifloxacin.

### Physician Assessment of Clinical Outcome

Overall, moxifloxacin was assessed as having “very good” or “good” clinical outcome by 94.0–95.3% of physicians across the regions. A higher proportion of physicians from Europe or the Middle East than from Asia Pacific rated moxifloxacin as “very good”. Conversely, a clinical outcome rating of “good” was more frequent among doctors in the Asia Pacific region (Table 5).

**Table 5:** Physicians’ assessment of clinical outcome.

Assessment	Total population (n = 3909)	Asia Pacific (n = 1421)	Europe (n = 1261)	Middle East (n = 1227)
Very good	2798 (71.6)	891 (62.7)	939 (74.5)	968 (78.9)
Good	897 (22.9)	463 (32.6)	246 (19.5)	188 (15.3)
Sufficient	100 (2.6)	44 (3.1)	30 (2.4)	26 (2.1)
Insufficient	88 (2.3)	17 (1.2)	41 (3.3)	30 (2.4)

### Safety of Moxifloxacin Treatment in ABS Patients

In total, there were 272 adverse events (AEs) in 159 of the 3909 patients (4.1%) in the safety population (Table 6, patient-based). The most frequently reported AEs were nausea, followed by diarrhoea, dizziness, headache, abdominal pain, abdominal pain upper and vomiting (Table 6). Patients could have experienced more than one AE. Adverse drug reactions (ADRs) were reported in 128 patients and the total number of ADRs was 272, the most common of which were nausea (n = 31), dizziness (n = 15) and diarrhoea (n = 16). Twenty one serious AEs (SAEs) were recorded in 6 patients (0.15%), the most common of which were hypersensitivity (n = 2), dyspnoea (n = 2) and rash (n = 2). Serious adverse drug reactions (SADRs) occurred in 5 patients, with the most common being reported as dyspnoea (n = 2), hypersensitivity (n = 2) and rash (n = 2). All SADRs resolved or improved by the end of the study period. No patient died during the study due to an AE.

**Table 6:** Incidence of adverse events (AEs) adverse drug reactions (ADRs), serious adverse events (SAEs) and serious adverse drug reactions (SADRs), and incidence of the most common AEs (occurring in ≥0.1% of patients) categorised by Medical Dictionary for Drug Regulatory Activities Preferred Term (MedDRA PT) in the valid for clinical outcome and safety population (N_total_ = 3909).

Event	Patients with AEs n (%)^a^
AEs	159 (4.1)
ADRs	128 (3.3)
SAEs	6 (0.2)
SADRs	5 (0.1)
**MedDRA PT AEs**	
Nausea	38 (1.0)
Diarrhoea	17 (0.4)
Dizziness	16 (0.4)
Headache	14 (0.4)
Abdominal pain	13 (0.3)
Abdominal pain (upper)	11 (0.3)
Vomiting	11 (0.3)
Abdominal discomfort	9 (0.2)
Insomnia	8 (0.2)
Tachycardia	6 (0.2)
Dyspepsia	6 (0.2)
Palpitations	5 (0.1)
Rash	5 (0.1)
Gastritis	4 (0.1)
Asthenia	4 (0.1)
Fatigue	4 (0.1)
Malaise	4 (0.1)
Myalgia	4 (0.1)
Dyspnoea	4 (0.1)
Rash pruritic	4 (0.1)

a Patients could have had more than one AE.

Investigators’ overall tolerability rating was “very good” or “good” in 3673 of 3909 patients (94.0%) (Table 7). There were regional differences in rating of tolerability. In Asia, fewer physicians rated moxifloxacin tolerability as “very good” and more physicians rated tolerability as “good” compared with those in Europe and the Middle East.

**Table 7:** Physicians’ assessment of tolerability.

Assessment	Total population (n = 3909)	Asia Pacific (n = 1421)	Europe (n = 1261)	Middle East (n = 1227)
Very good	2581 (66.0)	783 (55.1)	912 (72.3)	886 (72.2)
Good	1092 (27.9)	560 (39.4)	284 (22.5)	248 (20.2)
Sufficient	139 (3.6)	56 (3.9)	35 (2.8)	48 (3.9)
Insufficient	47 (1.2)	14 (1.0)	18 (1.4)	15 (1.2)

## Discussion

Rhinosinusitis is a global health problem which causes significant morbidity and leads to impaired quality of life, absence from school or work and substantial healthcare spending. Nevertheless, it does not require the systematic use of antibiotics and symptomatic therapy is sufficient in many instances. In ABS, however, antibiotics are necessary in some cases. In this context, early recognition of potentially problematic cases is important [28]. Intracranial complications of ABS are rare, but are associated with significant morbidity and mortality [29]–[31].

This observational study systematically collected data from thousands of patients with acute rhinosinusitis. The study findings suggest that there are differences between global regions in the clinical presentation, diagnosis and clinical course of ABS. The results for patient condition, frequency of severe rhinosinusitis, the frequency of severe main symptoms of acute rhinosinusitis, and the proportions of patients with 2 or more severe symptoms indicate that disease episodes were more severe in patients in Europe than in the other two regions. Because of this, the duration of symptoms before seeking medical advice was the longest in European patients than in those in Asia Pacific countries and the Middle East. This suggests that patients in different regions will tolerate the disease for different periods of time before seeking symptomatic relief. The lowest percentage of patients who had prior antibiotic therapy for the current episode was found in the Middle East (19.3%) compared with 21.8% of patients in Europe and 26.0% of patients in Asia. The duration of previous antibiotic therapy was the shortest in Asia Pacific (5.4±2.6 days), and was slightly longer in the Middle East (6.0±4.0 days), with the longest duration being documented in Europe (7.5±3.7 days). The more frequent use of topical decongestants or nasal preparations by patients in Europe compared with those in Asia Pacific which might have contributed to this finding; however, use of such medications in the Middle East was as common as in Europe. Furthermore, other therapies (e.g. sinus irrigation) were applied in approximately 8% of the patients with regional variations that might have had an impact on the clinical outcome of moxifloxacin. Consistent with the presence of more severe disease, acute rhinosinusitis had a greater and longer impact on the daily lives of patients in Europe than those in the other two regions. Although mean recovery times were similar across different regions, patients in the Asia Pacific region reported symptom resolution earlier on average than those in Europe or the Middle East, which may have been due to less severe disease at baseline.

Regional differences were seen in diagnostic procedures, with the highest average number of diagnostic measures used per patient and more frequent use of several procedures, particularly minimally invasive techniques, in Europe. Such differences may reflect resource availability, patient medical insurance arrangements and local practice guidelines. X-ray and CT are diagnostic tools that were used in a large proportion of patients for diagnosis, despite the fact that they do not differentiate between viral and bacterial sinusitis in uncomplicated cases. We can only speculate that, as in this study most of the investigators were ENT physicians, the severity of symptoms made it necessary to get a better understanding of the location of sinusitis, which was confirmed by multiple locations for a given patient and by the frequency of posterior sinusitis requiring further exploration to rule out other possible diagnoses. On the other hand, sinus puncture, although infrequently used, was more likely to be used in patients in European countries than in the other two regions. When patients fail on the chosen antibiotic and the presence of bacterial pathogen remains uncertain, this diagnostic tool is highly recommended when microbiological data is warranted for confirmation of causative pathogens, or even when patients suffer from severe symptoms associated with the presence of purulent exudates that cannot spontaneously evacuate [1].

In all regions, moxifloxacin provided good clinical outcome in a short treatment period, in part reflecting the excellent penetration of moxifloxacin into the sinuses [22], [23]. In addition, moxifloxacin was well tolerated with low numbers of discontinuations due to treatment-related adverse events. The current data support the results from randomised controlled clinical trials, which show that moxifloxacin is an effective treatment for ABS. The observation that low numbers of patients with mild disease were prescribed moxifloxacin indicates that it is generally being used in those patients who seem to be more appropriate for antibiotic treatment. The latest IDSA guidelines, however, suggest that moxifloxacin is reserved for patients who were clinical failures after first-line antibiotic treatments or who have known allergy to penicillin, and it should be reserved as second-line therapy for patients who are at risk of being infected with penicillin-non-susceptible *S. pneumoniae* [10]. *S. pneumoniae*, *H. influenzae*, *M. catarrhalis*, against which moxifloxacin has high in vitro activity, are the most prevalent species in ABS [8], [27] and also in other respiratory infections. However, susceptibility testing was not performed in this study as it is not required in routine clinical practice.

Previous studies have shown that moxifloxacin treatment of acute bacterial rhinosinusitis produces rapid symptom improvement with good tolerability [25], [26], and is at least effective as comparator antibiotics [32]–[35]. Numerous variables may affect the relevance of the results from randomised controlled trials to everyday clinical practice. The uncomplicated study design of a Phase IV observational study such as this requires no deviation from current medical practice for regulatory reasons. One limitation of observational studies is that it is not feasible to investigate all primary or secondary outcome parameters investigated in randomised controlled trials, since the latter enrol several hundreds of patients versus several thousands of patients in observational studies. Nevertheless, the inclusion of a large number of patients from a range of global locations treated according to local clinical practice and experience strengthens the evidence about the effects of, or the reactions to, a particular treatment.

An additional merit of large observational studies is that they allow identification of rare safety events that would not be seen in the relatively small populations usually enrolled in clinical trials. No unexpected safety events were seen in this study and no death occurred. Safety parameters were also assessed among patients with acute exacerbation of chronic rhinosinusitis without raising any concern regarding adverse events; no death occurred in this patient population.

Although no safety concerns were raised in the study regarding ABS patients or patients who were retrospectively excluded on the basis of having chronic rhinosinusitis, we have observed that the most common adverse events reported for ABS patients (shown in Table 6) occurred at slightly higher rates in the chronic rhinosinusitis patients (maximum difference <0.7% for each individual event). It is not unexpected because these patients may have an ongoing systemic inflammation and probably more comorbidities and co-medications; therefore, they could be more susceptible to treatment-emergent adverse events. However, as it was not the aim of the study to investigate patients with acute exacerbation of chronic rhinosinusitis, we currently cannot support such a statement in relation to patients’ characteristic and/or demographic data. Further appropriately designed clinical studies in this group would potentially clarify such proposition.

By the nature of its design, this non-comparative, non-controlled, non-randomised study has further limitations. Investigators were not required to confirm that the current episode of rhinosinusitis was bacterial in origin. Identifying causative bacteria in acute rhinosinusitis remains a challenging task for clinicians due to the nature of the surgical procedure it involves. However, in the current study the high proportion of patients with purulent nasal secretions and sinus puncture suggests the likelihood of bacterial aetiology. The sinuses involved in the current episode were not confirmed by imaging techniques in all cases, although this is consistent with current clinical guidelines [1]–[5], [10]. The study population was not stratified according to use of concurrent medications. Another limitation of this study is the lack of an active comparator antibiotic agent; it is difficult to run a controlled study involving thousands of patients and it would have been even more problematic to choose a single comparator agent that is acceptable for all countries where the study was undertaken.

### Conclusions

Acute bacterial rhinosinusitis continues to impact significantly on patients’ quality of life. Nevertheless, the results of this Phase IV observational, non-controlled study, along with previous Phase III, randomised controlled studies, suggest that moxifloxacin could be a valuable antibiotic choice for the treatment of ABS. However, differences between global regions in the clinical presentation, diagnosis and clinical course of acute bacterial rhinosinusitis highlight the need for better understanding of this common condition and its causes. Targeted physician education in all regions on the evolution of clinical practice guidelines and the importance of the adherence to such guidelines, including the value of diagnostic tools and more judicious antibiotic selection for therapy would have an enormous impact on the overall outcome after antibiotic treatment for this heterogeneous disease. This study provides a useful insight into the use of moxifloxacin in the treatment of acute bacterial rhinosinusitis in clinical practice. It indicates that when administered to patients with acute rhinosinusitis, moxifloxacin is broadly being used in accordance with existing clinical guidance and SmPC. Guidelines specify when moxifloxacin is a suitable option for treatment, and physicians should review their patients with this in mind. To our knowledge, this is the first observational study conducted in multiple regions allowing for comparisons in the clinical management of acute rhinosinusitis.

## Supporting Information

Checklist S1 STROBE Checklist for cohort observational studies.
(DOC)
